# Preparation and Characterization of Nanoparticles Made from Co-Incubation of SOD and Glucose

**DOI:** 10.3390/nano7120458

**Published:** 2017-12-19

**Authors:** Liping Cai, Chuntong Lin, Nannan Yang, Zhijie Huang, Song Miao, Xiaochao Chen, Jianru Pan, Pingfan Rao, Shutao Liu

**Affiliations:** 1 Institute of Biotechnology, Fuzhou University, Fuzhou 350108, China; lipingcaizz@sina.com (L.C.); linct666@163.com (C.L.); yangnannan@gelfeel.com (N.Y.); h603736570@163.com (Z.H.); chenxiaochao@gmail.com (X.C.); panjr@fzu.edu.cn (J.P.); pingfanrao@zjgsu.edu.cn (P.R.); 2 Teagasc Food Research Centre, Moorepark, Fermoy, Co. Cork, Ireland; Song.Miao@teagasc.ie

**Keywords:** superoxide dismutase, nanoparticle, Maillard reaction, cell uptake

## Abstract

The attractive potential of natural superoxide dismutase (SOD) in the fields of medicine and functional food is limited by its short half-life in circulation and poor permeability across the cell membrane. The nanoparticle form of SOD might overcome these limitations. However, most preparative methods have disadvantages, such as complicated operation, a variety of reagents—some of them even highly toxic—and low encapsulation efficiency or low release rate. The aim of this study is to present a simple and green approach for the preparation of SOD nanoparticles (NPs) by means of co-incubation of Cu/Zn SOD with glucose. This method was designed to prepare nanoscale aggregates based on the possible inhibitory effect of Maillard reaction on heating-induced aggregation during the co-incubation. Sodium dodecyl sulfate-polyacrylamide gel electrophoresis (SDS-PAGE) results indicated that the Maillard reaction occurred during the co-incubation process. It was found that enzymatically active NPs of Cu/Zn SOD were simultaneously generated during the reaction, with an average particle size of 175.86 ± 0.71 nm, and a Zeta potential of −17.27 ± 0.59 mV, as established by the measurement of enzymatic activity, observations using field emission scanning electron microscope, and analysis of dynamic light scattering, respectively. The preparative conditions for the SOD NPs were optimized by response surface design to increase SOD activity 20.43 fold. These SOD NPs showed storage stability for 25 days and better cell uptake efficacy than natural SOD. Therefore, these NPs of SOD are expected to be a potential drug candidate or functional food factor. To our knowledge, this is the first report on the preparation of nanoparticles possessing the bioactivity of the graft component protein, using the simple and green approach of co-incubation with glucose, which occurs frequently in the food industry during thermal processing.

## 1. Introduction

Superoxide Dismutase (SOD) plays a central role in the antioxidant system [1] because of its specific catalysis of the dismutation of the primary free radical—superoxide anion [2], which endows it with attractive potential in the fields of medicine and functional food. However, strategies based on the delivery of the natural form of SOD are limited because of its short half-life (~6 min) in circulation, as well as its poor permeability across the cell membrane and blood-brain-barrier [3,4]. The nanoparticle form of SOD may be able to overcome these limitations, and several groups have previously reported that nanoparticle-mediated delivery of SOD demonstrates a much better effect than the natural form of SOD on oxidative stress in different fields. For example, Vinod Labhasetwar’s group loaded SOD in poly (d,l-lactide-*co*-glycolide) NPs using a typical multiple-emulsion solvent-evaporation method, and found that the SOD NPs conferred better protective effects than controls against the oxidative stress of neutron and ischemia-reperfusion injury [3,4]. Vladimir R. Muzykantov et al. described the preparation of Protective Antioxidant Carriers for Endothelial Targeting (PACkET) through the controlled precipitation of oleate coated magnetite, SOD, and Pluronic F127 driven by CaCl_2_, and found SOD-loaded PACkET mitigated cytokine-induced endothelial pro-inflammatory activation and endotoxin-induced lung inflammation [5]. Kost et al. reported the preparation of SOD nanozyme by electrostatic coupling of SOD with a cationic block copolymer poly(l-lysine)-poly(ethylene-glycol) and covalent cross-linking with 3,3′-dithiobis (sulfosuccinimidylpropionate), and that the nanozyme was much more effective than the free enzyme in decreasing eye inflammation [6]. Karami et al. developed solid lipid nanoparticles (SLN) dispersions for enhancing SOD penetration across burned rat skin and protecting against environmental degradation by the cold homogenization method. They found that the percentage of activity by SLN dispersions through rat skin was 13 times more than that of the control [7]. Although a few preparative methods, for example polyketal microparticles, provide relatively safe and efficient delivery for SOD [8,9], most of these previous preparative procedures require either complicated operations, a variety of reagents—some of them even highly toxic—and low encapsulation efficiency or low release rate. These disadvantages have limited the applications of SOD NPs. Therefore, more alternative simple and green preparation methods are desirable.

Considering that protein is prone to aggregate under thermal processing—which disrupts various noncovalent interactions—it is possible to prepare nanoscale aggregates, as long as excessive aggregation is inhibited. On the other hand, an appropriate level of aggregation may maintain substantial bioactivity in the protein.

The Maillard reaction (MR) between the free amino group in proteins and the reducing-end carbonyl group in carbohydrates is a convenient and green modification that occurs frequently in nature and in food processing. It has been reported that egg lysozyme and phosvitin modified by MR with polysaccharides show good heat stability [10]. Our previous data indicated that Cu/Zn SOD co-incubated with glucose also exhibited improved thermal stability. Exposing the Cu/Zn SOD molecule to thermal processing will lead to the unfolding of the protein, which on cooling will induce aggregation due to the heat-induced disruption of various noncovalent interactions. This process may be reversible in SOD covalently modified by glucose, which inhibits the unfolded protein-protein interaction, whereas it may be irreversible in the SOD alone [11]. Therefore, it is possible for MR to inhibit excess aggregation of SOD under thermal processing.

The goal of this study was to investigate whether MR is useful for the preparation of SOD NPs by thermal processing, based on its inhibitory effect on aggregation. Therefore, SOD was simply co-incubated with glucose, a reducing monosaccharide existing widely in the human body and various foodstuffs. The Maillard reaction, morphological and physiochemical properties of the SOD NPs, condition optimization of the preparation of the NPs, and cell uptake efficacy of the reactants were all investigated in this study, which confirmed the feasibility of this design.

## 2. Results

### 2.1. Confirmation of the Maillard Reaction

**Figure 1:**
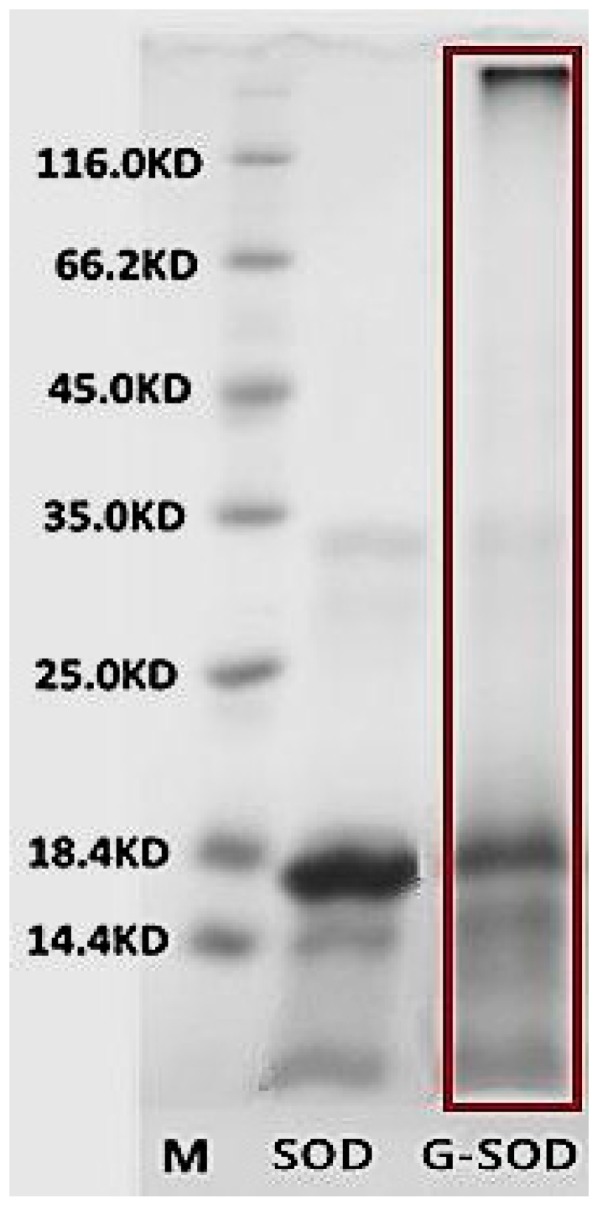
Sodium dodecyl sulfate-polyacrylamide gel electrophoresis (SDS-PAGE) pattern of superoxide dismutase (SOD) and the co-incubated reactant of Cu/Zn SOD and glucose (G-SOD).

The Maillard reaction of the co-incubated reactant of Cu/Zn SOD and glucose (hereinafter abbreviated as G-SOD) was investigated by SDS-PAGE. As shown in Figure 1, the un-incubated Cu/Zn SOD exhibited a main protein band with a molecular weight of about 16 KDa, while in the case of G-SOD, the density of this band decreased with an obvious tailing phenomenon, and a protein band with a molecular weight higher than 116 KDa also appeared with obvious tailing. These results indicated that the Maillard reaction occurred during the co-incubation process, and that some polymer aggregate of Cu/Zn SOD developed in the reactant [12].

### 2.2. Preparation and Characterization of Cu/Zn SOD Nanoparticles

**Figure 2:**
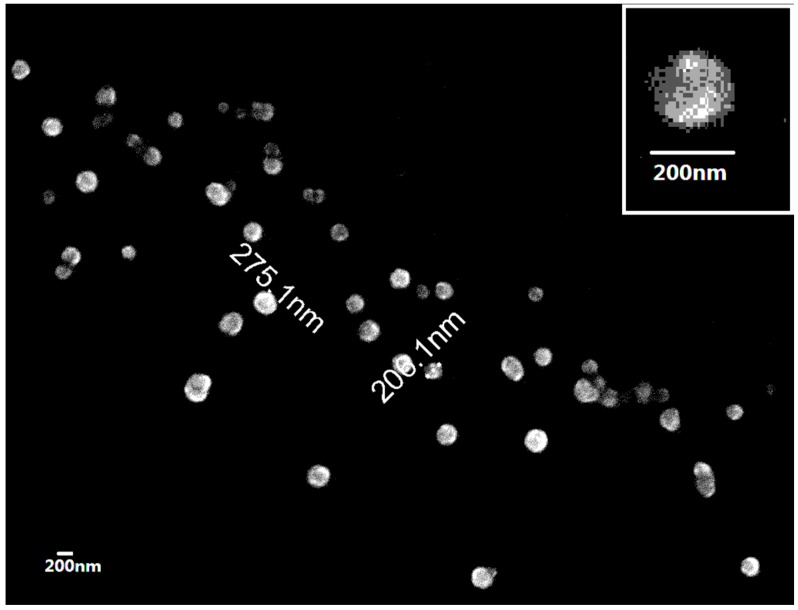
Nanoparticle pattern under field emission scanning electron microscope.

In order to prove our hypothesis that the MR—mentioned above in G-SOD—is useful for the preparation of Cu/Zn SOD nanoparticles, G-SOD was observed under the field emission scanning electron microscope (FESEM). As shown in Figure 2, the morphology of the nanoparticles in G-SOD is a spherical shape with a relatively uniform particle distribution between 100 and 300 nm. The inset is a magnification image of G-SOD nanoparticles, with a size close to 200 nm.

Both nanoparticle size and Zeta potential were measured using dynamic light scattering. It was found that the Zeta potential was −17.27 ± 0.59 mV and the average size was 175.86 ± 0.71 nm, which coincides with the nanoparticle pattern under FESEM observation.

The enzymatic activity of G-SOD was measured to be 1102.98 ± 31.37 U/mL. In order to verify whether this activity of SOD is derived from the SOD molecule or the free copper ion, the concentration of free copper ion in G-SOD was measured and the corresponding enzymatic activity was determined by measuring the activity of copper sulfate with the same concentration as that of the free copper ion. The results showed that the concentration of free copper ion was 2.90 ± 0.05 ppm with an enzymatic activity of 39.8 U/mL. This value only holds 3.62% of the enzymatic activity of G-SOD. Thus, the possibility that the enzymatic activity of G-SOD is from the free copper ions can be excluded, indicating that the residual activity of the protein molecule of Cu/Zn SOD is substantial after the co-incubation processing.

Next, a response surface design was used to optimize the preparation of G-SOD nanoparticles (NPs) with improved enzymatic activity, according to the principle of Box-Behnken combination design, as shown in Table 1 and Table 2. The optimal conditions were obtained as follows: the mole ratio of Cu/Zn SOD to glucose was 1:1, reaction temperature was 75 °C and reaction time was 45 min.

After optimization, the enzyme activity of G-SOD was 22,542.9 ± 879.1 U/mL; the average particle size and Zeta potential were 168.8 ± 0.6 nm and −13.66 ± 0.55 mV, respectively. These values were close to the predicted ones of the response surface design, in which enzymatic activity, average particle size, and Zeta potential of G-SOD were expected to be 22,969.8 ± 1305.6 U/mL, 192.5 ± 6.8 nm and −14.3 ± 1.6 mV, respectively.

The enzymatic activity of the product was increased 20.43 fold by the optimization of preparation conditions, mainly because the incubation temperature decreased from 100 to 75 °C, which reduces the thermal stress of Cu/Zn SOD molecule to be deep denaturation and aggregation. This phenomenon is consistent with our previous finding that 75 °C is the transiting temperature for the denaturation of Cu/Zn SOD [13].

**Figure 3:**
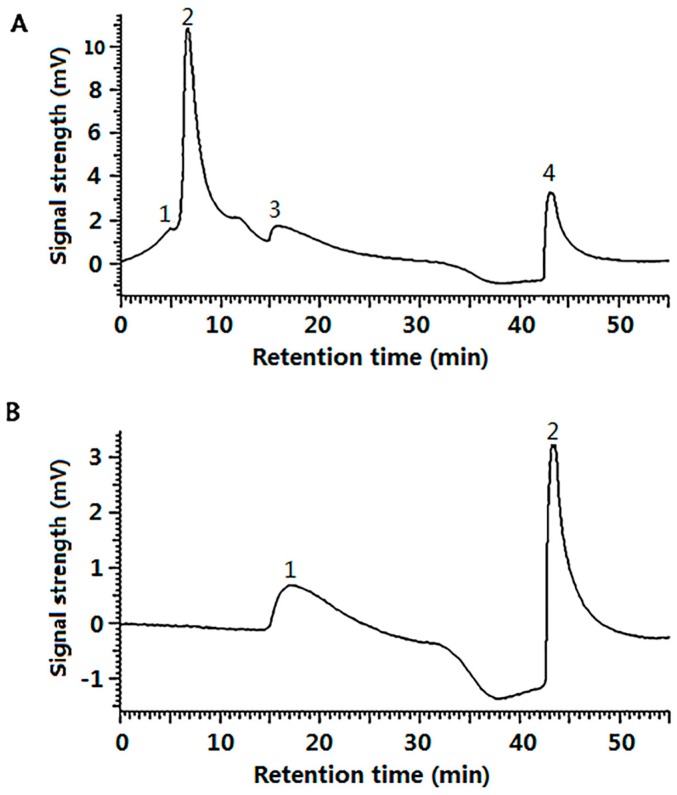
Gel filtration chromatography of G-SOD (**A**) and Cu/Zn SOD (**B**).

Furthermore, gel filtration chromatography of G-SOD was carried out to separate the aggregated polymer fraction from monomer or oligomer fraction of Cu/Zn SOD, so that confirming that NPs of G-SOD exhibited meaningful enzymatic activity. As to the reactants, four peaks appeared on the chromatogram with the retention times of 5, 6.7, 16 and 43.1 min, respectively (Peak 1–4, Figure 3A); Peak 1 and 4 were the peaks of void volume and total volume fraction, respectively. Thereby, G-SOD contained mainly fractions in Peak 2 and Peak 3. On the other hand, natural SOD showed only one peak (Peak 1) with a retention time of 17.1 min, other than the peak for total volume, on its chromatogram (Peak 2, Figure 3B). According to the gel filtration chromatography principle stating that fractions with higher molecular weight have a shorter retention time, it was deduced that the fraction of Peak 3 should be a monomer or oligomer Cu/Zn SOD, and Peak 2 be a polymer one. Based on the results of SDS-PAGE and nanoparticle analysis of G-SOD (Figure 1 and Figure 2), it was suggested that Peak 2 contained Cu/Zn SOD NPs. It was calculated according to their peak area in Figure 3A that the fraction of Peak 2 and Peak 3 comprised 66.1% and 33.9% of G-SOD, respectively. Therefore, the NPs were proposed to contribute most enzymatic activity to G-SOD, because the fraction of Peak 3 possibly only exerted 33.9% enzymatic activity of original sample, but G-SOD remained more than 95% of original activity. In another word, these results implied that NPs of G-SOD had meaningful enzymatic activity.

**Figure 4:**
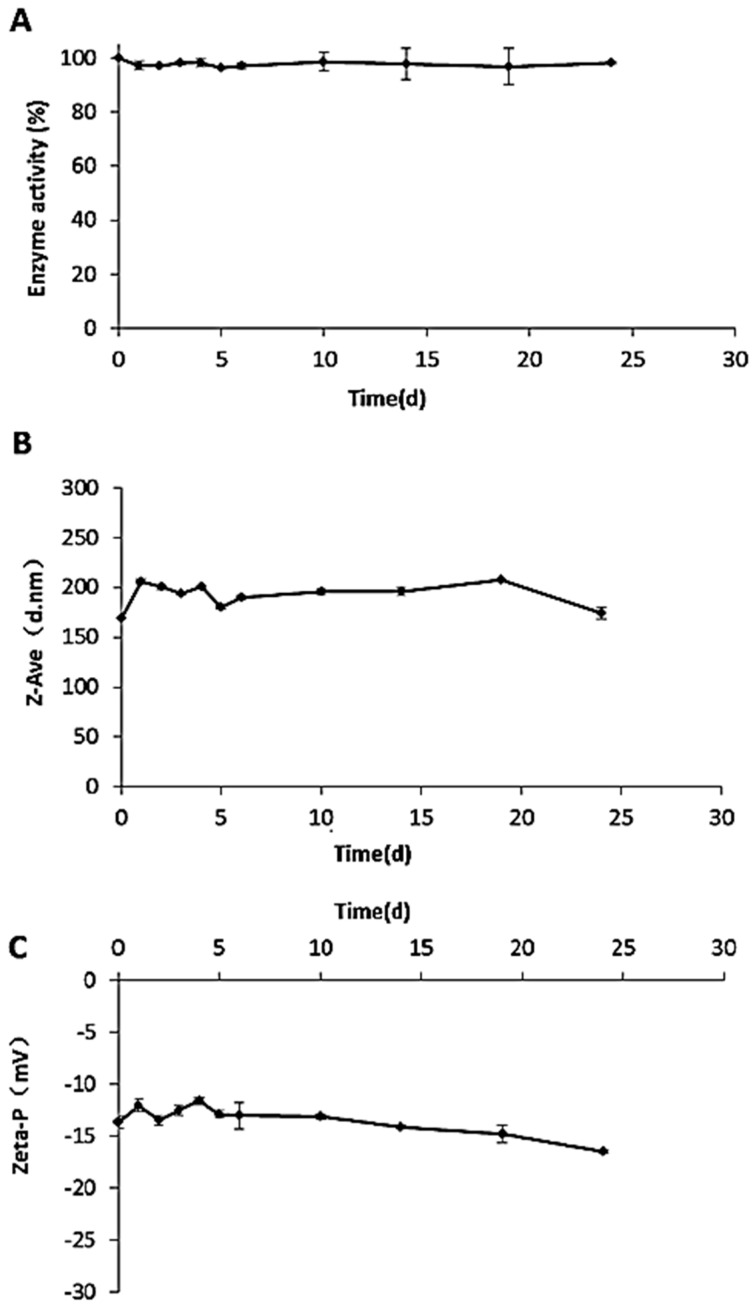
Effect of storage time on the enzymatic activity (**A**); average particle size (**B**) and Zeta potential (**C**) of G-SOD.

The storage stability of G-SOD NPs after optimization is important for future application. Therefore, the enzymatic activity, particle size and Zeta potential of the G-SOD were measured for 25 days at 4 °C, to evaluate its stability of storage. It was found that the enzymatic activity of G-SOD showed almost no decrease within 25 days, remaining at a high level with an average value of 22,652.5 ± 230.8 U/mL during the period of measurement (Figure 4A). The particle size and Zeta potential of G-SOD showed no obvious change within the detection time (Figure 4B, C), with a mean particle size of 192.5 ± 12.5 nm and a mean Zeta potential of −13.5 ± 0.33 mV. These data indicated that the optimized G-SOD could be stably present at 4 °C for at least 25 days. Moreover, there was also no marked change in the enzymatic activity, particle size or Zeta potential of the re-dissolved sample of lyophilized G-SOD (data not shown). All these results indicate that optimized G-SOD has good storage stability.

The above data confirmed that NPs of Cu/Zn SOD could be prepared by co-incubation with glucose and, more importantly, that these NPs retained substantial enzymatic activity and good storage stability.

### 2.3. The Cell Uptake Efficacy of G-SOD

Different concentrations of Cu/Zn SOD and G-SOD were co-cultured with alveolar macrophage cell line NR8383 for different times, and the SOD activity of the cell lysates was measured according to our previous paper [14]. SOD activity analysis of the cell lysates of the sample groups and control group involved in the formula of cell uptake efficacy, in order to calculate the delivery efficiency of G-SOD.

**Figure 5:**
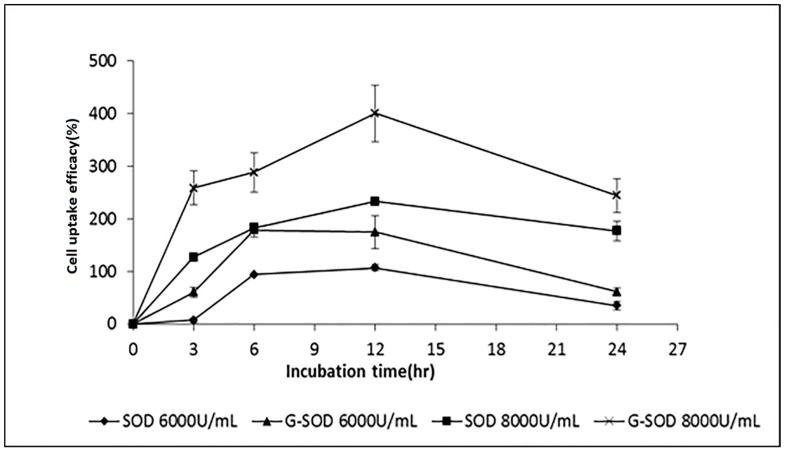
Cell uptake efficacy of Cu/Zn SOD and G-SOD in alveolar macrophages cell line NR8383.

It was found that alveolar macrophage cells themselves had a SOD activity of 44.39 U/mL (this value is defined as 100% in Figure 5), both types of SOD sample increased the intracellular SOD activity of alveolar macrophages, and the increment was related to concentration and co-culture time of the SOD samples (Figure 5). The enzymatic activity in the alveolar macrophages co-cultured with G-SOD in the same concentration was higher than that of the Cu/Zn SOD at the time points of 3, 6 and 12 h (*p* < 0.05). This indicated that the nano-form of Cu/Zn SOD found it easier to penetrate into the cells than natural Cu/Zn SOD. Additionally, it was also found that the uptake of SOD by alveolar macrophages was concentration dependent. In both the case of natural Cu/Zn SOD and G-SOD, the intracellular SOD activity of alveolar macrophages increased with the increment of the SOD concentration from 6000 to 8000 U/mL. The activity of SOD in alveolar macrophages also increased with the prolongation of co-culture time until 12 h or 6 h in the case of 6000 U/mL of G-SOD.

These results confirmed the hypothesis that the formation of Cu/Zn SOD nanoparticles by the co-incubation with glucose improved the cell uptake efficacy of SOD.

## 3. Discussion

In this study, nanoparticles of Cu/Zn SOD (G-SOD)—with an average particle size of about 175 nm—were prepared using a simple and green method of co-incubation with glucose. The optimized G-SOD retained meaningful enzymatic activity and good storage stability, and exhibited better cell uptake efficacy than natural SOD. It was found that the Cu/Zn SOD was modified by the Maillard reaction during co-incubation, which may have protected the Cu/Zn SOD from too much denaturation, aggregation and precipitation. Thereby, the reactant contained a large fraction of NPs with potential enzymatic activity; the life time of these nanoparticles in the buffer is more than 25 days (Figure 4) and about 6 h in-cell (Figure 5). These SOD-nanoparticles are stable and exert enzymatic activity directly, meaning that it is not necessary to release monomer SOD molecules in buffer, as shown in Figure 4A, though a portion of the SOD NPs that penetrates into the cell may release monomer SOD because of the interaction with the cell membrane. Therefore, the disadvantageous characteristics of Cu/Zn SOD, such as poor cell uptake efficacy and stability, were confirmed or are expected to be improved by the preparation of nanoparticles.

It is worth mentioning that the hypoglycemic effect of G-SOD and natural SOD were investigated on model rats of type I and type II diabetes, respectively. Based on the comparative data of body weight, blood glucose, oral glucose tolerance, tissue (containing heart, liver, kidney, pancreas, skeletal muscle) SOD levels, plasma insulin, adenosine monophosphate activated protein kinase and malondialdehyde (MDA) levels, it was found that G-SOD had a more pronounced hypoglycemic effect than natural SOD. Moreover, the administration of G-SOD dropped into the mouth of rat was found to have higher efficacy than did intragastric administration, indicating that these SOD NPs might be effective in the mouth and/or esophagus [15,16]. The effect of G-SOD was also tested on paraquat-induced oxidative stress in Hep G-2 cell. It was found that the efficacy of G-SOD on Hep G-2 cell repair was higher than that of natural SOD (data not shown).

These results suggest that the Cu/Zn SOD NPs could be a potential drug candidate or functional food factor. Compared with SOD NPs reported previously, these Cu/Zn SOD NPs possess some advantages. For example, the SOD NPs reported here are made by the moderate co-incubation of edible SOD and glucose; therefore, they are a kind of food-safe product with less need to worry about toxic components. Additionally, due to their natural composition of protein and monosaccharide, they could be supposed to be easy for digestion and excretion. Moreover, the higher efficacy of the administration of G-SOD dropped into the mouth of a rat than that of intragastric administration indicates that these SOD NPs might be effective in the mouth and/or esophagus before they are digested by pepsin and other digestive proteinases; thereby, they are useful as a factor of functional food. They might also have disadvantages, such as being liable to degradation; more undesirably, they are nonspecific, and therefore are difficult to use as a specific molecule for local oxidative stress, making them inferior to the antioxidant nanocarriers reported by Vladimir R. Muzykantov’s group [5,17].

More importantly, to our knowledge, this is the first report on the preparation of nanoparticles with the bioactivity of the graft component protein using the simple and green approach of co-incubation with glucose, a process frequently occurring in the food industry.

## 4. Materials and Methods

### 4.1. Materials

Cu/Zn SOD was purchased from the Institute of Tianjin Life Science and Application with a purity of 98%, glucose from Xi-Long Chemical Co., Shantou, China. Xanthine and 3-(4,5-dimethyl-2-thiazolyl)-2,5-diphenyl-2-H-tetrazolium bromide (MTT) were purchased from Sigma Co. (Milwaukee, WI, USA), xanthine oxidase from Roche Diagnostics Co. (Basel, CHE), streptomycin from Amersco Co. (Solon, OH, USA), cell culture medium and trypsin from Hyclone Co. (Logan, UT, USA), and the calf serum from Sijiqing Co. (Hangzhou, China). Hydroxylamine hydrochloride, Dimethyl sulfoxide (DMSO) and the rest of the commonly used drugs—such as anhydrous alcohol—were obtained from the National Medicine Group Chemical Reagent Co., Shanghai, China. All chemicals were of analytical grade.

### 4.2. Methods

#### 4.2.1. Sample Preparation

Cu/Zn SOD (1 mg/mL) was mixed with glucose with a mole ratio of 1:1. The mixture was heated at 60 °C by using a constant temperature water-bath (DK-8D, Shanghai Jing-Hong Laboratory Equipment Co., Shanghai, China) for 60 min, and then incubated at 100 °C for 60 min before being filtered through a 0.45-μm filter membrane. The filtrate of the reactant, noted as G-SOD, was aliquoted into the 2 mL tubes and stored at 4 °C for later experiments.

#### 4.2.2. Assay of Enzymatic Activity of SOD

Enzymatic product was evaluated with the hydroxylamine hydrochloride method using superoxide generated by xanthine oxidase [18,19] and calculated using an improved formula [20]. In this experiment, one unit of enzymatic activity (1 U) was defined as the amount of SOD that inhibits 50% of superoxide radical.

#### 4.2.3. Sodium Dodecyl Sulfate-Polyacrylamide Gel Electrophoresis (SDS-PAGE)

SDS-PAGE was carried out by using 12.5% of separation gel and 4% of concentrated gel according to Laemmli system [21]. Protein bands were stained by coomassie brilliant blue R-250.

#### 4.2.4. Observation of NP Morphology

Particle morphology was observed using field emission scanning electron microscopy (FESEM) with reference to a previous report [22]. SOD reaction liquid was put in 0.22 μm of cellulose acetate membrane and was fixated, dehydrated and dried. A layer of metal particles was coated onto the surface to ensure the conductive performance of the sample. A morphology image was obtained under scanning electron microscopy (S-4800, HITACHI, Tokyo, Japan).

#### 4.2.5. Measurement of Size and Zeta Potential of Nanoparticles

Dynamic light-scattering technology (DLS) has been widely used in the detection of the liquid dynamic behavior of large molecules, as well as micron-, submicron- and nano-scale particles [23]. It was also used to measure the size and Zeta potential of NPs in this study. Specific test parameters were as follows: Material—Polystyrene latex; Dispersant—Water; Viscosity—0.8872; Temperature—25 °C; Equilibration time—120 s; Cell type—DTS0012 disposable sizing curette; Measurement Angle—90°; Measurement duration—automatic; Number of measurements—3 (Zetasizer Nano ZS90, Malvern Instrument Co., Ltd, (Malvern, UK).

Zeta potential is often used to characterize the stability of the glue system [24]. The instrument testing parameter settings were as follows: Cell type—DTS1060C (clear the disposable Zeta Cell); Model—Smolushowski; other parameters were the same as for the particle size measurement.

#### 4.2.6. Determination of the Concentration of Copper Ion

Inductively Coupled Plasma Mass Spectrometry (ICP-MS) (Agilent-7500A, Agilent Technologies Co., Ltd., Santa Clara, CA, USA) system was used to analyze the concentration of copper ion, as described previously [25]. The analysis parameters of instrument were as follows: The radio frequency power—1390 w; Sampling depth—7 mm; the carrier gas flow rate—1.09 L/min; atomizing chamber temperature—2 °C; Integration time—0.1 s; Repeat measurement times—3.

#### 4.2.7. Optimization the Preparation of NPs by Response Surface Design

Factors such as incubation time, temperature, ratio of Cu/Zn SOD to glucose, which may influence enzymatic activity, and particle size and Zeta potential of NPs, were selected as the response values. Preparation technology could be optimized by response surface analysis (RSA) according to the principles of Box-Behnken combination design [26], as in Table 1 and Table 2.

#### 4.2.8. Gel Filtration Chromatography of G-SOD

Twenty microliters of sample were subjected to a Super SW3000 column (TSK-GEL, TOSOH, Tokyo, Japan) equipped in a HPLC system (D-2000 Elite, HITACHI, Tokyo, Japan). The Super SW3000 was equilibrated with buffer A (0.2 M phosphate buffer, pH 7.8) at a rate of 0.25 mL/min, eluted by a 30 min linear gradient of buffer A from 0% to 100% (*v*/*v*) of buffer B (I grade water) from 100% to 0%, followed by buffer A for 30 min. The absorbance of eluates was monitored at 280 nm. Peaks were identified by comparing their retention time.

#### 4.2.9. Assay of Cell Uptake Efficacy of G-SOD

Alveolar macrophages cell line NR8383 was used to investigate the cell uptake efficacy of G-SOD and Cu/Zn SOD. Cells were suspended in F-12K medium and adjusted to a density of 1 × 10^6^ cells/mL. A 1-mL aliquot of this cell suspension was seeded to a well of a 6-well plate, followed by incubation for 24 h. Each well was washed with phosphate-buffered saline (PBS, 1 mL) and 1mL SOD samples with a concentration of 6000 U/mL or 8000 U/mL, 1 mL medium without serum were added subsequently. After 3, 6, 12 and 24 h of incubation, the medium was discarded and the surface of the cells was washed with PBS to remove Cu/Zn SOD or G-SOD. The wells at 0 h, to which were added the same volume of PBS, instead of the SOD sample, were used as control. The cells were digested with 0.25% trypsin and centrifuged to remove trypsin. After washing twice with PBS, 0.36 mL of sterile water and 40 μL of 1% Triton X-100 were added, and then the cells were disrupted with a shaking mixer. The SOD activity was determined after the cell disrupted solution was collected and stored at 4 °C for 30 min. Cell uptake efficacy was calculated by a formula as follows:

Cell uptake efficacy = [(SOD activity of the sample group − SOD activity of the control group)/SOD activity of the control group] × 100%

#### 4.2.10. Stability in Storage

It is quite important for application that the sample has certain stability in storage. After the sample was prepared, it was dispensed into a 2-mL centrifuge tube and placed at 4 °C. A sample was taken at days 0, 1, 2, 3, 4, 5, 6, 15, 20, and 25, and the enzymatic activity, particle size and Zeta potential were measured after filtration by a 0.45 μm filter. Stability in storage was evaluated according to the changes of measured enzymatic activity, particle size and zeta potential. The smaller of the change implies more stable of the NPs.

## Figures and Tables

**Table 1:** Factors and their levels used in a Box-Benhnken design.

Factors	−1	0	+1
Ratio	1	2	3
Temperature (°C)	60	75	90
Time (min)	30	45	60

**Table 2:** Response surface optimization design.

Number	Ratio	Temperature (°C)	Time (min)
1	1	75	60
2	2	90	30
3	2	75	45
4	2	75	45
5	1	90	45
6	2	75	45
7	2	60	30
8	3	75	60
9	3	60	45
10	1	75	30
11	2	75	45
12	1	60	45
13	2	90	60
14	2	75	45
15	3	90	45
16	2	60	60
17	3	75	30

## References

[1] Segui J., Gironella M., Sans M., Granell S., Gil F., Gimeno M., Coronel P., Pique J.M., Panes J. (2004). Superoxide dismutase ameliorates TNBS-induced colitis by reducing oxidative stress, adhesion molecule expression, and leukocyte recruitment into the inflamed intestine. J. Leukoc. Biol..

[2] Mccord J.M., Edeas M.A. (2005). SOD, oxidative stress and human pathologies: A brief history and a future vision. Biomed. Pharmacother..

[3] Reddy M., Labhasetwar V. (2009). Nanoparticle-mediated delivery of superoxide dismutase to the brain: An effective strategy to reduce ischemia-reperfusion injury. Faseb J. Off. Publ. Fed. Am. Soc. Exp. Biol..

[4] Reddy M.K., Wu L., Kou W., Ghorpade A., Labhasetwar V. (2008). Superoxide dismutase-loaded PLGA nanoparticles protect cultured human neurons under oxidative stress. Appl. Biochem. Biotechnol..

[5] Hood E.D., Chorny M., Greineder C.F., Alferiev I., Levy R.J., Muzykantov V.R. (2014). Endothelial targeting of nanocarriers loaded with antioxidant enzymes for protection against vascular oxidative stress and inflammation. Biomaterials.

[6] Kost O.A., Beznos O.V., Davydova N.G., Manickam D.S., Nikolskaya I.I., Guller A.E., Binevski P.V., Chesnokova N.B., Shekhter A.B., Klyachko N.L. (2016). Superoxide Dismutase 1 Nanozyme for Treatment of Eye Inflammation. Oxid. Med. Cell. Longev..

[7] Karami M.A., Zadeh B.S.M., Koochak M., Moghimipur E. (2016). Superoxide Dismutase-Loaded Solid Lipid Nanoparticles Prepared by Cold Homogenization Method: Characterization and Permeation Study Through Burned Rat Skin. Jundishapur J. Nat. Pharm. Prod..

[8] Lee S., Yang S.C., Heffernan M.J., Taylor W.R., Murthy N. (2007). Polyketal microparticles: A new delivery vehicle for superoxide dismutase. Bioconjug. Chem..

[9] Fiore V.F., Lofton M.C., Roser-Page S., Yang S.C., Roman J., Murthy N., Barker T.H. (2010). Polyketal microparticles for therapeutic delivery to the lung. Biomaterials.

[10] Nakamura S., Ogawa M., Nakai S., Kato A., David D.K. (1998). Antioxidant Activity of a Maillard-Type Phosvitin? Galactomannan Conjugate with Emulsifying Properties and Heat Stability. J. Agric. Food Chem..

[11] Cai L., Qiu D., Liu S., Pan J., Yang N., Zhao D., Zhang H., Fu T., Miao S. (2017). Effect of Coexisting Glucose on the Thermal Stability of Superoxide Dismutase. J. Chin. Inst. Food Sci. Technol..

[12] Pirestani S., Nasirpour A., Keramat J., Desobry S. (2017). Preparation of chemically modified canola protein isolate with gum Arabic by means of Maillard reaction under wet-heating conditions. Carbohydr. Polym..

[13] Yang Z., Zhang C., Ao W., Cai Z., Liu S., Rao P. (2011). Purification of Recombinant Human Cu/Zn SOD Expressed in Pichia Yeast and Its Stability Characterization. J. Chin. Inst. Food Sci.Technol..

[14] Pan J., He H., Su Y., Zheng G., Wu J., Liu S., Rao P. (2016). GST-TAT-SOD: Cell Permeable Bifunctional Antioxidant Enzyme—A Potential Selective Radioprotector. Oxid. Med. Cell. Longev..

[15] Zhao D., Cai L., Fu T., Zhang H., Liu S., Rao P. (2016). Hypoglycemic effect of glycated SOD on alloxan-induced diabetic rats. J. Chin. Inst. Food Sci. Technol..

[16] Zhao D. (2016). Hypoglycemic Effect of Maillard Reaction Products of Superoxide Dismutase in Diabetic Rats. Master’s Thesis.

[17] Hood E., Simone E., Wattamwar P., Dziubla T., Muzykantov V. (2011). Nanocarriers for vascular delivery of antioxidants. Nanomedicine.

[18] Guo J., Chen Y., Yuan B., Liu S., Rao P. (2011). Effects of intracellular superoxide removal at acupoints with TAT-SOD on obesity. Free Radic. Biol. Med..

[19] Elstner E.F., Heupel A. (1976). Inhibition of nitrite formation from hydroxylammoniumchloride: A simple assay for superoxide dismutase. Anal. Biochem..

[20] Zhang C., Bruins M.E., Yang Z.Q., Liu S.T., Rao P.F. (2016). A new formula to calculate activity of superoxide dismutase in indirect assays. Anal. Biochem..

[21] Laemmli U.K. (1970). Cleavage of Structural Proteins during the Assembly of the Head of Bacteriophage T4. Nature.

[22] Fathollahipour S., Abouei Mehrizi A., Ghaee A., Koosha M. (2015). Electrospinning of PVA/chitosan nanocomposite nanofibers containing gelatin nanoparticles as a dual drug delivery system. J. Biomed. Mater. Res. Part A.

[23] Khlebtsov B.N., Khlebtsov N.G. (2011). On the measurement of gold nanoparticle sizes by the dynamic light scattering method. Colloid J..

[24] Bhattacharjee S. (2016). DLS and zeta potential—What they are and what they are not?. J. Control. Release.

[25] Khan N., Jeong I.S., Hwang I.M., Kim J.S., Choi S.H., Nho E.Y., Choi J.Y., Park K.S., Kim K.S. (2014). Analysis of minor and trace elements in milk and yogurts by inductively coupled plasma-mass spectrometry (ICP-MS). Food Chem..

[26] Dandekar D.V., Jayaprakasha G.K., Patil B.S. (2008). Hydrotropic extraction of bioactive limonin from sour orange (*Citrus aurantium* L.) seeds. Food Chem..

